# Identifying Burnout in Pediatric Clinicians Using Wearable Biometric Data: Development and Feasibility Study

**DOI:** 10.2196/104511

**Published:** 2026-10-29

**Authors:** Thomas B Ayres, George E Quaye, Harpreet S Gill, Addison Pritchett, Catherine Koertje, James S Leeder, Brooke L Fridley, Tiffany Addington

**Affiliations:** 1 Center for Wellbeing, Children's Mercy Hospital, 2401 Gilham Rd, Kansas City, MO, 64108, United States, 1 9182324404; 2 School of Medicine, University of Missouri–Kansas City, Kansas City, MO, United States; 3 Division of Health Services & Outcomes Research, Children's Mercy Hospital, Kansas City, MO, United States; 4 Research Informatics & Data Science, Children's Mercy Hospital, Kansas City, MO, United States; 5 Research & Sponsored Projects, Children's Mercy Hospital, Kansas City, MO, United States; 6 Children’s Mercy Research Institute, Kansas City, MO, United States; 7 Division of Urgent Care, Children's Mercy Hospital, Kansas City, MO, United States

**Keywords:** burnout, health care workforce, wearable device, machine learning, Maschlach Burnout Inventory, pilot study, pediatrics

## Abstract

**Background:**

Addressing the burnout epidemic among health care providers remains one of the foremost challenges of modern health care systems. Past researchers have turned to passively gathered biometric data from wearable devices to identify key variables that predict burnout. However, this has resulted in limited success.

**Objective:**

This pilot and feasibility study aimed to test the feasibility and accuracy of developing an Extreme Gradient Boosting machine learning model to classify health care providers as being at higher or lower risk of burnout based on their biometric data.

**Methods:**

We conducted a 3-month pilot and feasibility study where health care providers self-reported burnout every 6 weeks and wore a Garmin Venu 3 smartwatch to collect continuous biometric data. Participants were recruited from 3 pediatric urgent care sites and an inpatient hospitalist division of a pediatric health care system. We had a final sample of 41 participants who met our inclusion criteria requiring completion of burnout measurements at 2 of the 3 time points and having worn the watch for at least 70% of the study period. Participants were asked to wear the Garmin watch continuously over the course of the study. Burnout self-report surveys were delivered via email. Our main outcome was burnout as measured using the Maslach Burnout Inventory–Human Services Survey. The measure consists of 3 subscales that together constitute burnout: emotional exhaustion, depersonalization, and reduced personal efficacy.

**Results:**

First, we found evidence of feasibility for wearable watches to be used in situ for health care providers to gather data passively for the purposes of classifying burnout. A total of 82% (51/62) of participants met our criteria of wearing the watch 70% of the time, with 66% (41/62) having corresponding burnout scores to be included in analyses. Second, our machine learning model achieved strong overall performance in identifying risk of burnout, with strong sensitivity and specificity, demonstrating that biometric data collected using wearable devices can accurately predict burnout.

**Conclusions:**

Our evidence of feasibility offers promising results for a passive method of data collection to be used for classifying burnout risk in other health care settings. Furthermore, the strong performance of our machine learning model provides a foundational step toward improving an organization’s ability to respond and mitigate burnout through thoughtful intervention strategies.

## Introduction

Understanding and reducing burnout in health care providers (HCPs) continues to be a paramount challenge in medicine [1]. Burnout is characterized by emotional exhaustion, depersonalization, and feelings of low personal accomplishment [2]. Research on burnout in HCPs often relies on self-report surveys that are time-consuming and reflective. In addition, HCPs often do not recognize when they are developing burnout until it has occurred, when its impact on them personally and professionally is readily apparent [3,4]. There is an established correlation between medical professional burnout and diminished quality of health care delivery, leading to medical errors and increased health care costs [5-7].

Burnout is typically not monitored continuously, and the interval between assessments delays the opportunity for organizations and employees to intervene. There is a need for methods to predict impending burnout in HCPs, with the goal of delivering interventions during this “at-risk” time point to prevent burnout. Researchers have endeavored to improve the measurement of burnout by looking for biometric markers that could be continuously measured via a wearable device and, therefore, reduce delay in the notification of burnout risk and subsequent intervention for HCPs [8].

Recent studies have explored wearable-derived physiological metrics such as heart rate, sleep patterns, and activity levels as indicators of stress and burnout risk [8-11]. When looking for direct predictive relationships between a biometric and self-reported burnout, researchers have reported no reliable biometrics, with the exception of average resting heart rate, which was found to be higher in residents experiencing burnout than those not experiencing burnout [8,11]. However, researchers note that biometrics such as heart rate and heart rate variability (HRV) are connected to more momentary acute indicators of stress [8]. While biometric data from wearable devices have not been connected to burnout and resilience, wearable biometric devices were implemented in a randomized clinical trial setting, and those in the intervention arm showed lower burnout and higher resilience at 6 months after the intervention [12]. These findings suggest that there is an important but nuanced relationship between biometrics and well-being.

This prior work has focused on identifying individual biometrics rather than leveraging the combined predictive value of multimodal biometric data using machine learning approaches [8]. This gap limits the ability to capture complex physiological signatures underlying burnout as well as whether the biometrics can be used to determine whether someone is more at risk of being burned out [8]. We contribute to this growing body of literature by conducting a pilot and feasibility study examining whether a supervised machine learning technique using the Extreme Gradient Boosting (XGBoost) algorithm to build a predictive model using daily biometric data can correctly classify whether an HCP is at risk of burnout.

Some researchers suggest that the reason for not finding a connection between biometric data and burnout is that the length of measurement may be too short, noting that very few studies have a duration greater than 12 months [8]. However, in practical application, requiring 12 months of biometrics to identify burnout creates barriers to addressing individuals’ needs in a timely manner and poses challenges to measurement adherence. Recent research on the reliability and accuracy of biometric wearable devices suggests that data can be meaningfully interpreted after short periods [13]. Therefore, we propose using machine learning to reliably predict individuals at risk of burnout with as little as 3 months of biometrics. As a result, the initial goal of this pilot study focused on determining the feasibility of using wearable devices to monitor risk of burnout in HCPs in acute care and inpatient pediatric settings within a 3-month time frame.

## Methods

### Study Participants and Procedures

The study population of HCPs was selected from 3 pediatric urgent care sites and a pediatric hospital within Children’s Mercy in Kansas City, Missouri. Participants were enrolled between October 2024 and January 2025. Data collection took place between October 2024 and May 2025. Inclusion eligibility criteria included full-time or part-time status as a Children’s Mercy physician, advanced practice provider, or nurse within the urgent care and/or hospitalist departments and age of 18 years or above. Participants of all races, ethnicities, and sexes were eligible. Those Children’s Mercy employees with primary employment outside the designated study departments working as “moonlighters” were excluded. All enrolled participants were provided with a Garmin Venu 3 smartwatch to enable real-time collection of biometric data. The Garmin Venu 3 was chosen for the pilot study as it captures continuous biometric data and has shown evidence of accuracy [13]. Participants were encouraged to wear the smartwatch continuously for 3 months. Participants were also asked to complete the Maslach Burnout Inventory–Human Services Survey (MBI-HSS) every 6 weeks starting at baseline to provide a standard for burnout identification to allow for assessment of the prediction model for burnout.

### Ethical Considerations

This study, numbered STUDY00003352, was approved as exempt from full review by the Children’s Mercy Institutional Review Board.

### Burnout Classification Measures

We used the MBI-HSS to measure burnout in our study [2]. The MBI-HSS uses 3 subscales to measure burnout: emotional exhaustion, depersonalization, and reduced personal accomplishment. Example items include “I feel emotionally drained by my work” (emotional exhaustion), “I have become more insensitive to people since I’ve been working” (depersonalization), and “Through my work, I feel that I have a positive influence on people” (reduced personal accomplishment). Burnout is most often viewed as existing on a continuum from “not at all burned out” to “extremely burned out” [2]. However, this continuum approach creates a challenge when trying to identify which of the HCPs should be targeted with interventions for reducing burnout because there are no universally accepted clinical cutoffs for burnout [14].

Given our primary objective of identifying HCPs at higher risk of burnout, median scores were calculated from the distribution of each MBI-HSS subscale across the study cohort and used as thresholds to classify participants into higher- and lower-risk groups. These thresholds were not intended to represent clinical diagnostic cutoffs, nor were they derived from individual participant baseline trends over time. As no universally accepted clinical cutoffs exist for burnout, median-based classification was selected to facilitate more balanced class distributions for supervised machine learning. Consequently, burnout classification in this study should be interpreted as a relative indicator of burnout risk within the study population rather than a definitive clinical diagnosis.

### Biometric Data

Participants wore the Garmin Venu 3 smartwatch, which recorded HRV, sleep score, stress level, step count, and distance walked throughout the study period. All variables were obtained as day-level summaries exported from the Garmin platform. Heart rate was derived from wrist-based photoplethysmography, a widely used method in consumer wearables that shows reasonable agreement with electrocardiography under resting and sleep conditions [15,16].

Sleep was summarized using Garmin’s nightly sleep score, a composite measure reflecting sleep duration, estimated sleep stages, movement, and physiological signals during sleep. Prior validation studies indicate that wearable-derived sleep metrics, including those from Garmin devices, are appropriate for characterizing longitudinal patterns rather than precise sleep staging [16,17].

Stress level was derived from HRV patterns and reflects autonomic nervous system activation, with higher values indicating greater physiological stress. HRV is a well-established marker of stress and recovery and has been validated for use in population-level and longitudinal analyses [18,19]. Step count and distance walked were recorded as daily activity totals. All biometric measures served as raw inputs for subsequent analysis.

### Analytical Approach

#### Feature Engineering, Variable Selection, and Missing Data

To capture temporal variation in biometric data, we computed weekly summary statistics for all features, including mean, minimum, maximum, SD, median, IQR, and skewness for each participant over the 12 weeks. Weekly summary statistics were computed using all available daily observations within each week, with missing daily values handled via complete-case aggregation (ie, summary statistics were computed using available observations only). As participants had varying start and end dates and some of the data collected were sporadic rather than continuous, we aligned the time windows relative to each participant’s enrollment date. The weekly summaries were subsequently reshaped into a wide, cross-sectional feature matrix, with each statistic indexed by week number. In total, this process generated 756 features (12 weeks × 7 summary measures × 9 features) for each participant for further analysis.

Given the high-dimensional nature of the dataset with 756 features and only 41 observations, we faced a classic p ≫ n scenario where the number of predictors far exceeded the number of participants in the study. To reduce dimensionality and mitigate the risk of overfitting, we applied univariate feature screening using both parametric 2-tailed *t* tests and nonparametric 2-tailed Wilcoxon rank-sum tests, with features retained if they demonstrated statistical significance in either test. This dual approach ensured robustness to distributional assumptions, which is particularly important given the heterogeneity and sparsity of the biometric data. A liberal threshold (*P*<.10) was selected to reduce the risk of excluding potentially informative predictors in this high-dimensional, low-sample setting. This approach aligns with established strategies for variable selection in high-dimensional settings [20]. After the initial univariate screening, a total of 122 features were retained for modeling the risk of burnout outcome. The retained features spanned multiple biometric domains, with most derived from stress and heart rate–related measures, followed by sleep score and activity metrics. Across domains, retained predictors primarily reflected measures of central tendency and extreme values, with additional contributions from variability-based statistics and a smaller number of skewness measures.

#### Machine Learning Modeling

To develop predictive models for the 3 MBI-HSS subscales and the overall burnout risk classification, we used supervised machine learning techniques through the XGBoost algorithm [21]. XGBoost is a highly efficient and scalable ensemble learning method that builds additive models in a forward, stagewise manner. It is particularly well suited for structured data and high-dimensional settings, offering strong performance through regularization, handling of missing values, and built-in feature importance metrics. This choice was motivated by the need for a robust algorithm capable of capturing complex relationships in a dataset with a large number of features and a relatively small sample size. XGBoost’s ability to prevent overfitting and its flexibility in tuning make it an ideal candidate for modeling biometric data in burnout prediction. Due to the limited number of participants, we were unable to implement a training and testing framework. To ensure robust performance estimation and reduce the risk of overfitting, models were trained using 5-fold cross-validation (CV) [22]. During each fold, the model was trained on 80% of the data and evaluated on the held-out 20%. Predicted class probabilities for the positive outcome were saved for all observation-fold combinations. Prior to model fitting, all predictor variables were centered and scaled to standardize feature distributions across participants.

While not all outcome variables exhibited class imbalance, for those that did, we incorporated the ROSE (Random Over-Sampling Examples) approach within the CV framework to avoid information leakage [23]. ROSE sampling was applied within the training CV folds only and not to the held-out CV folds or data. Model performance was evaluated using the area under the receiver operating characteristic curve (AUC), sensitivity, and specificity. Discriminative performance was assessed using CV by aggregating out-of-fold predicted probabilities across all folds and constructing a single receiver operating characteristic curve, yielding a pooled cross-validated estimate of the AUC.

CIs for the AUC were calculated using the DeLong method [24]. Optimal classification thresholds were determined using the Youden *J* statistic [25] to balance sensitivity and specificity. All analyses were conducted in R (version 4.4.1; R Foundation for Statistical Computing), with machine learning analyses completed using the *caret* and *xgboost* R packages. To allow for other researchers to explore the deidentified data, an R Shiny application was also created and is available [26].

## Results

### Feasibility

We enrolled 62 HCPs across the 3 urgent care sites and pediatric hospital medicine within Children’s Mercy in Kansas City. Feasibility was defined as the ability of HCPs to wear the device at least 70% of the days over the study period, with a day being counted if the participant had at least 500 steps with the device on for more than 70% of the day (16 hours). Survey response feasibility was defined as a response rate on average of at least 70% for each of the MBI-HSS surveys administered at each scheduled time point, with 70% of participants completing 2 of the 3 surveys. Feasibility for both objectives was successful with 82% (51/62) meeting device feasibility and 73% (45/62) meeting the survey feasibility. Therefore, we found evidence of feasibility in that HCPs who were working in their clinical settings at the time of the study were able to wear the device long enough to obtain meaningful data. After exclusion of HCPs who did not meet feasibility thresholds, 66% (41/62) remained and were included in our analysis. Characteristics of the HCPs included in the study are provided in Table 1. Characteristics for the entire sample are included in the Shiny app [26]. When asked about the difficulty or challenges of wearing the biometric device, participants reported a few reasons that could be tied to nonadherence. Participants with smaller-sized wrists found our specific device, the Garmin Venu 3, to be a bit too bulky and interfere with exercise and movement. Some participants reported skin irritation due to the bands or metal. Others reported that previous habits of not wearing a watch during the day or at night presented some difficulties in terms of remembering to wear the watch or getting used to wearing it.

**Table 1.** Characteristics of clinicians by burnout status. Summary of clinical, demographic, and professional characteristics among clinicians, presented overall and stratified by burnout status (N=41).

Characteristics	Overall, n (%)	Burnout, n/N (%)		*P* value^a^
		Yes (n=32)	No (n=9)	
What is your clinical specialty?				.005
Hospital medicine	17 (41.5)	17/17 (100)	0 (0)	
Urgent care	24 (58.5)	15/24 (62.5)	9/24 (37.5)	
What is your age (y)?				.11
25-34	7 (17.1)	5/7 (71.4)	2/7 (28.6)	
35-44	16 (39)	13/16 (81.3)	3/16 (18.8)	
45-54	9 (22)	9/9 (100)	0 (0)	
55-64	9 (22)	5/9 (55.6)	4/9 (44.4)	
To what gender identity do you most identify?				.30
Female	33 (80.5)	27/33 (81.8)	6/33 (18.2)	
Male	8 (19.5)	5/8 (62.5)	3/8 (37.5)	
Race				.70
Asian	4 (9.8)	4/4 (100)	0 (0)	
Black or African American	1 (2.4)	1/1 (100)	0 (0)	
Latino or Hispanic	1 (2.4)	1/1 (100)	0 (0)	
White	35 (85.4)	26/35 (74.3)	9/35 (25.7)	
I am a (n):				.70
Advanced practice provider (NP)	10 (24.4)	7/10 (70)	3/10 (30)	
Physician (MD, DO, MBBS)	31 (75.6)	25/31 (80.6)	6/31 (19.4)	
Years of practicing medicine (y post medical professional degree)				.70
0-5	5 (12.2)	3/5 (60)	2/5 (40)	
11-15	10 (24.4)	9/10 (90)	1/10 (10)	
16-20	5 (12.2)	4/5 (80)	1/5 (20)	
≥21	12 (29.3)	9/12 (75)	3/12 (25)	
6-10	9 (22)	7/9 (77.8)	2/9 (22.2)	
What is your work schedule?				>.99
Full time	31 (75.6)	24/31 (77.4)	7/31 (22.6)	
Part time	10 (24.4)	8/10 (80)	2/10 (20)	
Which urgent care site do you work at primarily (anchor site)?				<.001
Adele hall	17 (41.5)	17/17 (100)	0 (0)	
Blue valley	6 (14.6)	4/6 (66.7)	2/6 (33.3)	
East	9 (22)	8/9 (88.9)	1/9 (11.1)	
North	9 (22)	3/9 (33.3)	6/9 (66.7)	

a Fisher exact test.

### Prediction of HCP Burnout

For those with demonstrated adherence to watch wear, we created a predictive model for burnout risk using biometric data. We classified HCPs as having an outcome of higher or lower risk based on whether their MBI-HSS composite and individual subscale burnout scores were above or below a median threshold (Table 2 and Multimedia Appendix 1). Using biometric features, the model demonstrated strong discriminative performance in identifying HCPs at higher risk of burnout. The model also achieved strong overall performance in identifying risk of burnout, with an AUC of 0.88 (95% CI 0.70‐1.00), indicating excellent discriminative power (Table 3). Sensitivity was 91%, reflecting high accuracy in detecting true cases, whereas specificity was 89%, showing reliable exclusion of nonburnout cases (Figure 1A). We also found robust results for model performance predicting individual dimensions of burnout (Table 3 and Figure 1B-1D).

We assessed which biometric features were most predictive of burnout from our machine learning model. We found that maximum heart rate, the skewness of distance walked in meters, and minimum heart rate were among the most predictive features in the model. However, variable importance reflects contribution to model prediction and does not imply causal relationships. A full list of variable importance measures is shown in Figure 2.

**Table 2.** Summary statistics for burnout subscale scores. Descriptive statistics for the 3 Maslach Burnout Inventory–Human Services Survey subscales (emotional exhaustion, cynicism [depersonalization], and professional efficacy), summarized across all clinicians (N=41).

Measure (min-max)	Mean (SD)	Median (IQR)	Range
High emotional exhaustion (0-6)	3.44 (1.12)	3.78 (1.44)	1.22-5.33
High cynicism (0-6)	1.72 (0.92)	1.80 (1.40)	0.20-3.80
Low professional efficacy (0-6)	3.72 (1.33)	4.13 (2.38)	1.25-5.88

**Table 3.** Performance metrics for machine learning models predicting burnout outcomes.

Outcome	AUC^a^ (95% CI)	Sensitivity (95% CI)	Specificity (95% CI)
Emotional exhaustion	0.89 (0.79-0.99)	0.86 (0.64-0.97)	0.85 (0.62-0.97)
High cynicism	0.88 (0.76-1.00)	0.90 (0.70-0.99)	0.85 (0.62-0.97)
Low professional efficacy	0.85 (0.73-0.97)	0.82 (0.60-0.95)	0.84 (0.60-0.97)
Overall burnout	0.88 (0.70-1.00)	0.91 (0.75-0.98)	0.89 (0.52-1.00)

a AUC: area under the receiver operating characteristic curve.

**Figure 1.**
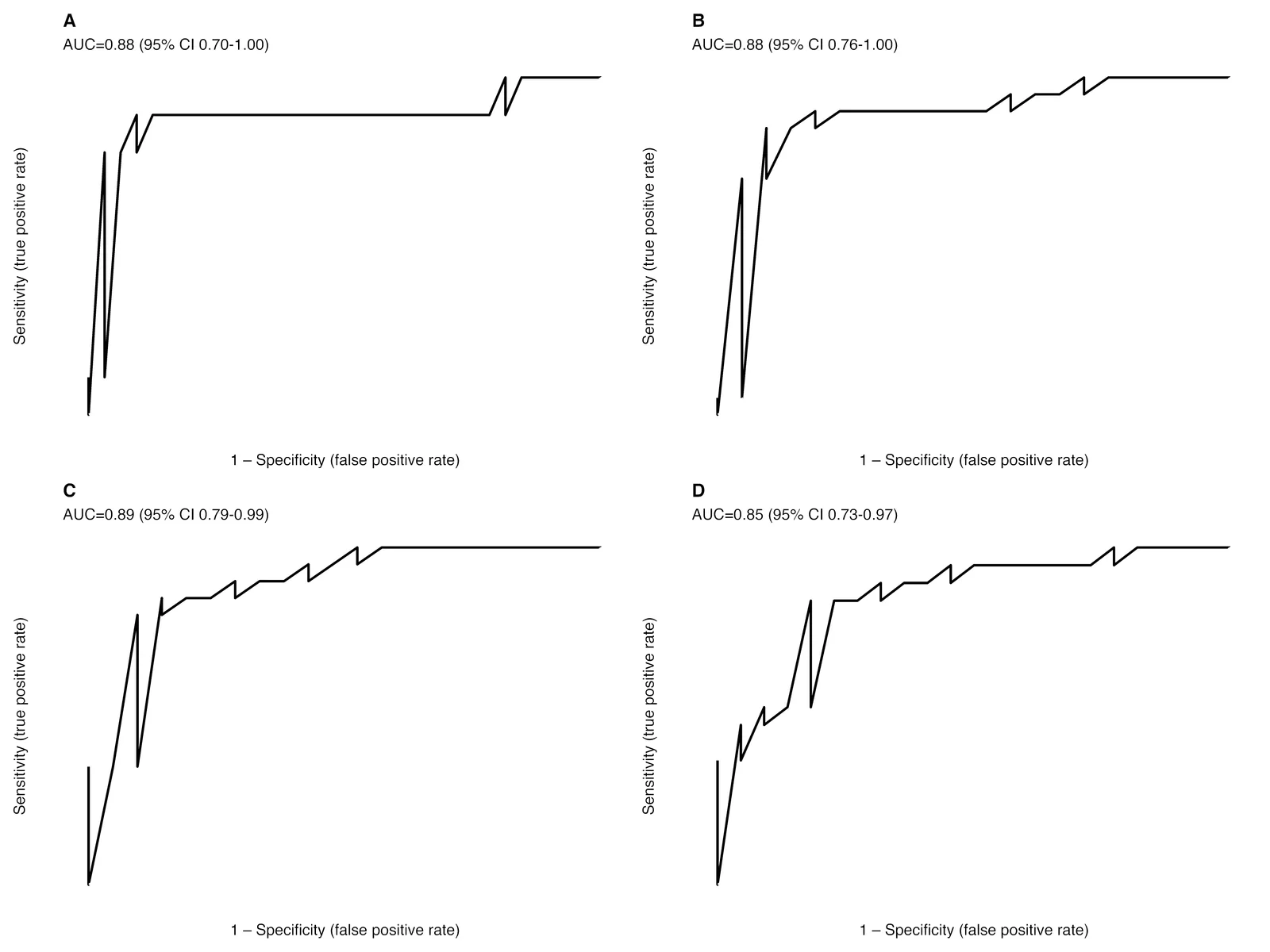
Receiver operating characteristic (ROC) curves for the 4 supervised machine learning models trained to classify burnout risk among health care providers using 12 weeks of biometric summary features: (A) overall burnout, (B) high cynicism (depersonalization), (C) high emotional exhaustion, and (D) low professional efficacy. Each panel includes the ROC curve (blue line), the diagonal line representing chance classification (gray dashed line), and the area under the ROC curve (AUC) with 95% CIs. Higher AUC values indicate stronger model discrimination.

**Figure 2.**
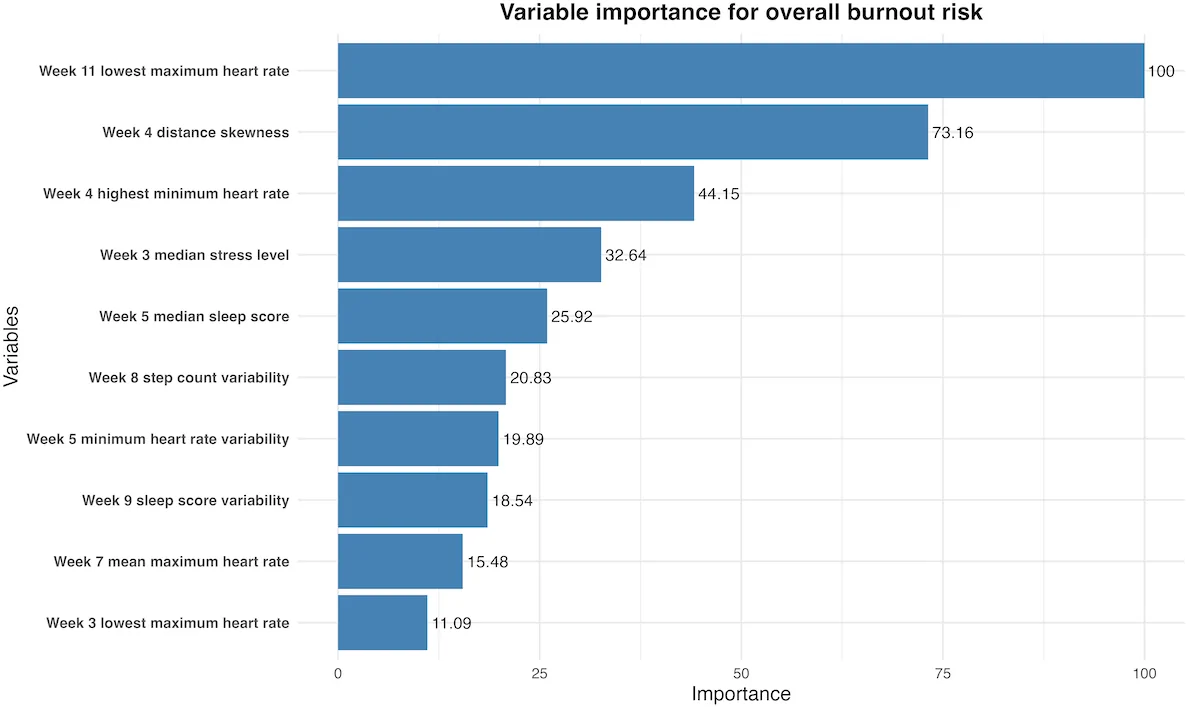
Relative importance of the top biometric features contributing to the model predicting overall burnout risk. Bars indicate each feature’s contribution to model discrimination based on 12-week biometric summaries. Maximum heart rate, skewness in walking distance, and minimum heart rate were the strongest contributors, followed by stress score, sleep score, and step count variability measures.

## Discussion

### Principal Findings

The goals of this pilot study were twofold: (1) determine the feasibility of using smartwatch technology to monitor burnout and (2) test whether the biometric data can be used to predict burnout risk in HCPs within a 3-month time frame. To answer these questions, we used a wearable smartwatch device (ie, Garmin Venu 3) to gather physiological biometrics to predict which HCPs were at the highest risk of burnout. We found evidence that a machine learning model that incorporates all the biometric features over a 3-month period is accurate in categorizing HCPs as high risk vs low risk of burnout. Rather than focusing on individual biometrics, our study expands the utility of connecting wearable technology to burnout by examining biometrics as a dynamic sum to identify HCPs who are at higher risk of experiencing burnout [8].

### Strengths and Limitations

Strengths of our study include our ability to accurately classify HCPs as being at high or low risk of experiencing burnout with only 3 months of biometric measurements. This is substantially shorter than the periods that are advocated for in current research (>12 months) [8]. Future research would still, however, benefit from collecting data for a longer period. This would enable our machine learning model to be tested across various 3-month periods, better assessing the generalizability of our finding of strong model performance with just 3 months of data. Furthermore, we introduced machine learning as a methodological innovation to the study of HCP biometrics and burnout. This expands our ability to assess specific predictors of burnout toward a holistic examination of human experience and multiple biometric measures that can predict burnout. Future research could build on these findings by also predicting a positive indicator of well-being in resilience.

There are limitations to this study. Our small, opportunistic sample comprised participants belonging to homogenous demographic groups. For example, all participants were HCPs in a pediatric setting. Most of the study participants were female, and only 2 participants identified as being of non-White race. Our study was focused on pediatric providers, and our findings suggest that both pediatric advance practice providers and physicians were accurately reflected in our analysis. More research is needed to establish the applicability of this methodology for nonpediatric HCPs and a more diverse sample of genders and races. For example, burnout may be driven in part by social determinants of health or other psychological and economic factors; however, with such a homogenous sample, we are unable to parse out any of those effects in our data. Other specific causes of burnout were not identified either. While our study is not in scope for answering questions regarding specific causes of burnout, the ability to identify who is most at risk of experiencing burnout regardless of the cause is an important foundation on which to build future research. Looking forward, future studies will benefit by expanding this feasibility and pilot study to a larger and more diverse sample of HCPs to appropriately account for demographic contributors to burnout and generalize to a broader sample of HCPs.

In addition, our analysis is based on cross-sectional data and does not predict or assess change in burnout over time as the scope of this study was limited to determining whether biometric data from a wearable device could predict burnout risk at a future stage. Future research would benefit from examining the ability of biometric data to predict burnout changes over time using a within-subject change perspective.

Another limitation of our machine learning model is that our approach to capturing the individual variation across time during the 3 months by collapsing to aggregated week scores could not account for the variation in timing of study initiation. The nominal “week 1” refers to the first week of enrollment for every HCP in the study, but week 1 could take place on different calendar weeks of the year, potentially bringing HCPs into the study facing different organizational, seasonal, and personal contexts. Future research could benefit from either ensuring that all participants begin in the same week or comparing results between a “week of study” approach as we did and a “week of year” approach aligning by calendar date. Our results provide motivation to further explore the ability to predict HCP burnout from biometric data measured using wearable devices. Because model performance was estimated using CV within a relatively small cohort, the reported results should be interpreted as preliminary and may overestimate performance in independent populations. External validation in larger and more diverse cohorts will be necessary to establish the reproducibility and generalizability of the proposed prediction framework.

### Interventions Using Biometric Data

Furthermore, the feasibility evidence from our study supports developing interventions for HCPs identified as being at higher risk of burnout. Prior studies have examined the implementation of wearable devices as a self-feedback look in a randomized clinical trial structure and the use of smartphone apps as well-being interventions [12,27]. These studies found evidence of improved well-being and decreased stress as evidenced by higher resilience and lower burnout [12,27]. These findings are promising but leave a gap to be filled using biometric data to initiate the intervention. Our results show a promising indicator that using biometric data to initiate an intervention may be possible. Feedback regarding the feasibility of using wearables to collect biometric data also highlighted an opportunity for studying more models and types of biometric devices, such as alternative watch models or rings, to improve comfort for HCPs with varying physical statures.

One approach would be to provide recommended organizational resources with the burnout risk alert. Connecting the burnout risk identification capability of biometric data with targeted intervention stands to make a major contribution to the field of HCP burnout by enabling a timely and targeted intervention. However, there are important ethical considerations when developing interventions based on personal biometric data. Previous researchers have focused on conducting interventions at the individual level, with all accountability and access to data remaining with the individual [12,27]. This intervention structure creates a barrier between individuals’ personal biometric data and organizational oversight. However, there is a strong sentiment that well-being work needs to move toward organizational-based interventions that can have a greater impact on the aspects of work that influence burnout [28-30]. Incorporating the use of biometric indicators for organizational interventions introduces a complexity that must be addressed with careful consideration. Protecting the privacy of individuals, preventing a culture of surveillance, and degree of accountability for organizations regarding allowing HCPs to deliver care when aware that they are not at their best are just some of the aspects that future researchers will need to consider and incorporate into any studies on potential organizational interventions based on biometric data.

### Conclusions

In conclusion, wearable devices have value in understanding HCP burnout. We found evidence that rather than looking for specific biometrics driving burnout, a more holistic examination of biometrics using machine learning can accurately identify those individuals at higher risk of burnout. Researchers should continue examining factors related to HCP burnout, prediction of burnout, and development of interventions to test in clinical settings.

## Supplementary material

10.2196/104511 Multimedia Appendix 1 Counts of clinicians meeting the threshold criteria for high cynicism, high emotional exhaustion, low professional efficacy, and overall burnout.

## Abbreviations

AUC: area under the receiver operating characteristic curve
CV: cross-validation
HCP: health care provider
HRV: heart rate variability
MBI-HSS: Maslach Burnout Inventory–Human Services Survey
ROSE: Random Over-Sampling Examples
XGBoost: Extreme Gradient Boosting
